# Identify-Isolate-Inform: A Tool for Initial Detection and Management of Measles Patients in the Emergency Department

**DOI:** 10.5811/westjem.2015.3.25678

**Published:** 2015-03-18

**Authors:** Kristi L. Koenig, Wajdan Alassaf, Michael J. Burns

**Affiliations:** * University of California, Irvine, Department of Emergency Medicine, Orange, California; † University of California, Irvine, Department of Emergency Medicine and Department of Medicine, Division of Infectious Diseases, Orange, California

## Abstract

Measles (rubeola) is a highly contagious airborne disease that was declared eliminated in the U.S. in the year 2000. Only sporadic U.S. cases and minor outbreaks occurred until the larger outbreak beginning in 2014 that has become a public health emergency. The “Identify-Isolate-Inform” tool will assist emergency physicians to be better prepared to detect and manage measles patients presenting to the emergency department. Measles typically presents with a prodrome of high fever, and cough/coryza/conjunctivitis, sometimes accompanied by the pathognomonic Koplik spots. Two to four days later, an erythematous maculopapular rash begins on the face and spreads down the body. Suspect patients must be immediately isolated with airborne precautions while awaiting laboratory confirmation of disease. Emergency physicians must rapidly inform the local public health department and hospital infection control personnel of suspected measles cases.

## INTRODUCTION

The 2014 Ebola outbreak, a public health emergency originating in West Africa, as well as the emergence of Middle East Respiratory Syndrome and pandemic influenza, are stark reminders that we must prepare our healthcare systems for emerging infectious diseases (EIDs).^1^ The public health threat includes not only novel diseases, but also the reemergence of existing diseases that were previously well controlled, such as measles in the U.S. In 2014, 644 cases of measles from 27 states were reported to the U.S. Centers for Disease Control (CDC), the greatest number reported since the endemic disease was declared eradicated in the year 2000. From January 1 to March 6, 2015, CDC reported 173 people from 17 states and the District of Columbia to have measles, with most related to a multi-state outbreak linked to an amusement park in southern California.^2^

Measles is one of the most transmissible diseases in existence, with at least a 90% infection rate in susceptible populations.^3^ The incubation period ranges from 7–21 days, and humans are the only natural host. Measles can be contagious four days prior to the onset of rash and, in fact, is most contagious prior to rash manifestation. Measles can mimic influenza, croup, bronchiolitis or pneumonia before the rash occurs. It can be transmitted from surfaces and air for up to two hours after an infected person leaves a room. Yet most clinicians in industrialized countries have never seen even a single case of measles.

Following a brief review of measles, this paper describes the novel 3I tool, initially developed for Ebola virus disease,^4^ as adapted for use in the initial detection and management of measles patients in the emergency department (ED).

### Clinical Presentation

#### Symptoms and signs

**Figure 1:**
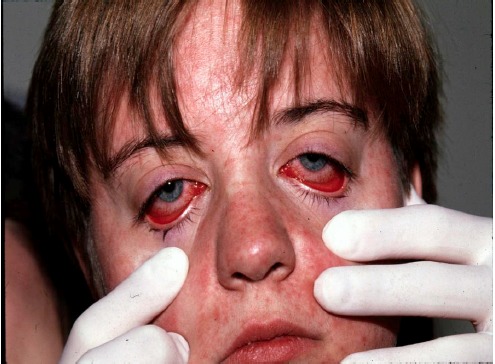
Nonpurulent conjunctivitis and facial rash of measles one day after rash began. Photo used with permission of Michael J. Burns, MD.

Measles classically presents with a high fever (often >104°F [40°C]), generally of 4–7 days in duration. This initial sign occurs after an incubation period of 1–2 weeks following exposure (average 10–12 days). During this prodromal phase, a classic triad of cough, coryza and conjunctivitis (the “3 Cs”) is often present.^5^ Patients may have photophobia. The eyes have a characteristic appearance, typically showing erythema of the palpebral conjunctiva with nonpurulent discharge (Figure 1) and sometimes periorbital edema. Patients may also report malaise, myalgias, anorexia, and diarrhea. Adults often develop transient hepatitis.^6^

**Figure 2:**
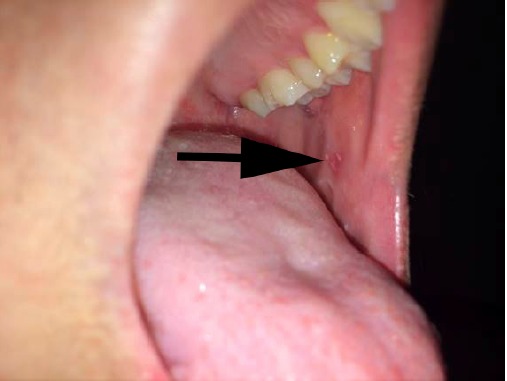
Koplik spots. Photo used with permission of Michael J. Burns, MD.

Koplik spots, when seen, are pathognomonic of measles (Figure 2). If present, they manifest 1–2 days prior to the rash and last for 3–5 days. They appear as bluish-gray enanthema (“small grains of sand”) on a red base and are typically seen on the buccal mucosa opposite the second molars. Therefore, it is essential to have proper lighting to visualize them. During a measles outbreak, after donning appropriate respiratory protection, emergency physicians (EP) should carefully assess the oropharynx in patients presenting with non-specific viral syndromes and assess for the presence of Koplik spots.

**Figure 3:**
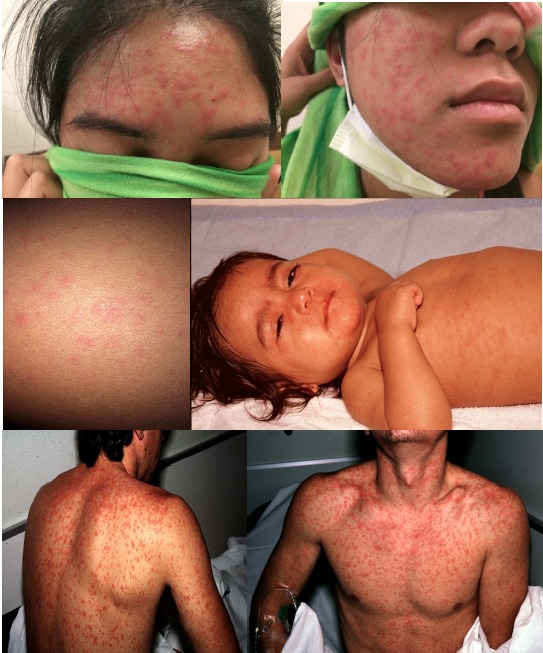
Measles rash. Photos used with permission of Michael J. Burns, MD.

**Figure 4:**
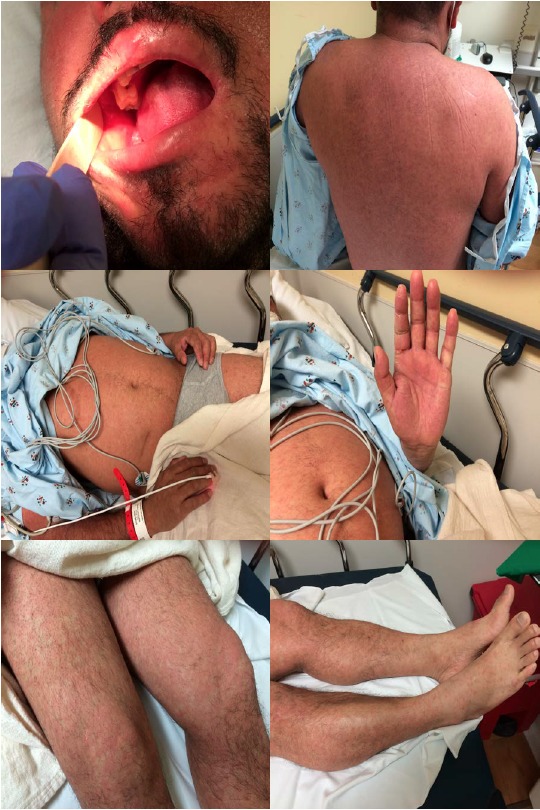
Measles rash and Koplik spots in an adult male. Photos used with permission of Michael J. Burns, MD.

The rash of measles generally erupts about 14 days after exposure, which is usually 2–4 days after onset of symptoms. Unlike rashes of some infectious diseases that start on the lower extremities or trunk, the rash of measles begins on the face and progresses cephalocaudally to the torso and extremities. Thus, assessing the pattern of rash evolution is essential to identify measles patients. Erythematous macules and papules coalesce into patches and plaques within about 48 hours (Figure 3). Petechia and ecchymosis can also be seen. By the time a rash develops, within 1–2 days, patients will be ill appearing. After 5–7 days, the exanthem begins to fade, forming coppery-brown hyperpigmented patches that may desquamate. The rash initially disappears at the location where it first appeared. The rash can be more difficult to detect on dark-skinned patients (Figure 4).

#### Manifestations of infection

Disease manifestations are often more severe in children under five and adults over 20 years of age. Patients who are immunocompromised may present atypically and may not develop a rash. During a measles outbreak, clinicians should advise patients with viral syndromes who are being discharged from the ED to monitor for appearance of a rash, especially one that first appears on the face. If a rash develops, children or adult patients should avoid public places and seek immediate medical advice.

#### Atypical Measles

People who received vaccinations between 1963 and 1967 with the original killed-virus measles vaccine may have incomplete immunity and present with milder symptoms. During those years, this vaccine was only administered to U.S. children at about one year of age, so persons presenting in 2015 with “atypical measles” would be 48–52 years of age. A prodrome of fever, headache, abdominal pain and myalgias can be subclinical. In these atypical presentations, the rash can be macular, vesicular, petechial, or urticarial and can begin on the hands and feet and spread centripetally. When atypical measles was first reported in the late 1960s and early 1970s, it was often mistaken for Rocky Mountain Spotted Fever.

In patients who receive post-exposure prophylaxis with serum immunoglobulin, a modified variety of measles can occur with similar but milder signs and symptoms and an incubation period of up to 21 days.

### Risk Factors

The population most at risk for measles are those persons who are exposed, but not immunized, or with inadequate immunity. This includes infants too young to receive immunizations (under 12 months), children whose parents have declined immunization, travelers from countries where immunization rates are low, and immunocompromised patients.

### Diagnosis

When measles is suspected, the clinician should collect separate swabs of the throat and nasopharynx using viral culture swabs and contact the local health department for real-time polymerase chain reaction (RT-PCR) testing. Serum can also be obtained and sent for measles-specific IgM antibody, but the test may be negative early in the course of disease. Some local health departments may also request additional testing of serum and urine for RT-PCR testing. According to the CDC, there are no data supporting routine checking of serum antibody titers, particularly given the fact that they have notable false negative result rates.^7^

### Complications and Special Populations

Pregnant women and other persons with deficiencies in cell-mediated immunity are at increased risk for serious complications, including primary measles giant cell pneumonia with respiratory failure. Spontaneous abortion and premature delivery have been described. However, there is no increased risk for congenital anomalies as has been reported in other diseases contracted during pregnancy, such as toxoplasmosis, rubella, cytomegalovirus, herpes simplex, and human immunodeficiency virus.

The vast majority of patients with measles who are well-nourished (not vitamin A deficient) recover; however, measles can lead to several complications and even death.^5^,^8^ While certain groups of patients are at high risk for complications, even previously healthy children can become severely ill and require hospitalization.

The following are well-described complications of measles:

- 1 in every 10 children develops bacterial otitis media (can lead to permanent hearing loss)
- 1 in every 20 children develops bacterial pneumonia
- 1 in every 1000 children develops acute encephalitis (often resulting in permanent brain damage)
- Unknown frequency of measles giant cell pneumonia in pregnant and other immunocompromised patients
- 1 in every 1000 children dies (from respiratory and neurologic complications)
- Febrile seizures are also seen

In addition, a rare late complication (7 to 10 years after measles infection) is subacute sclerosing panencephalitis (SSPE), a fatal degenerative disease of the central nervous system characterized by behavioral and intellectual deterioration and seizures. Permanent blindness may also result from measles infection.

### Transmission and Personal Protective Equipment

Measles is transmitted by the airborne route. It can be contracted for up to two hours from the air or from airborne particles on surfaces in a room that was occupied by a measles patient. In addition to standard precautions, all practitioners, even if immunized, who enter a room with a suspected or confirmed measles patient should wear fit-tested N95 respirators or equivalent respiratory protection.

### Differential Diagnosis

**Figure 5:**
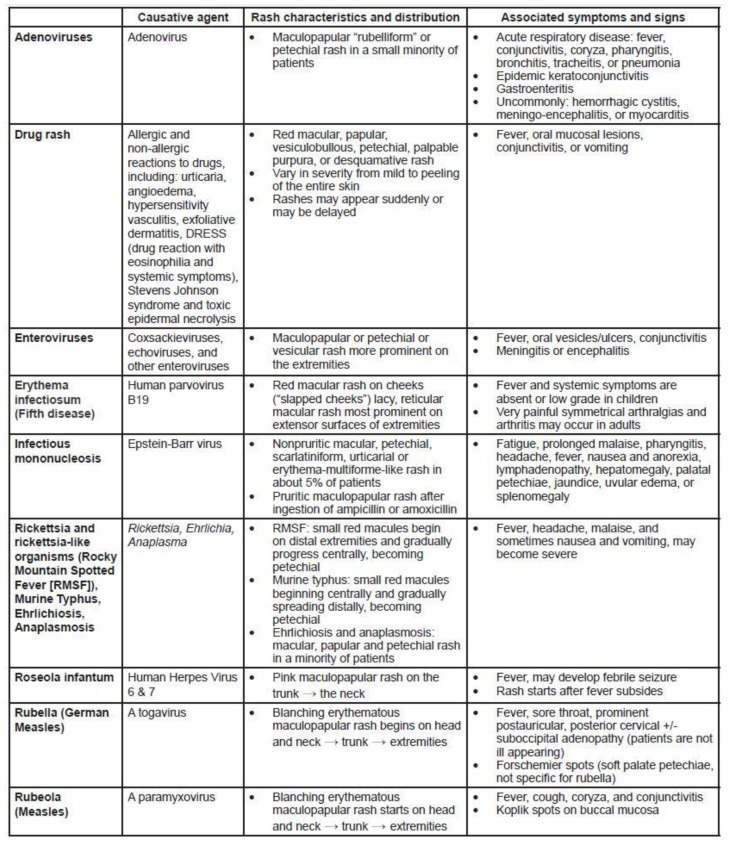

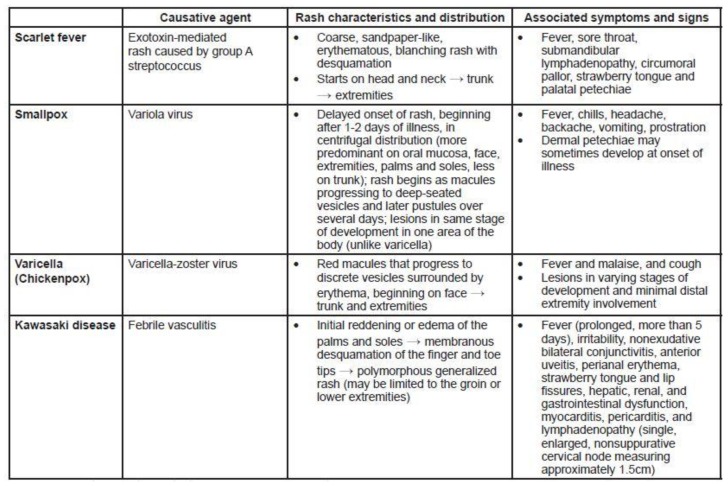
Pediatric exanthems that may mimic measles.

Prior to onset of rash, measles can mimic influenza, croup, bronchiolitis, other viral illnesses, and pneumonia. Once the rash develops, particularly with accompanying fever, other entities, including common childhood diseases, are in the differential diagnosis. Characteristics of these diseases help to distinguish them from measles (Figure 5). In addition to alternate infections, EPs must distinguish the rash of measles from other acute rash presentations with systemic symptoms, including allergic drug reactions.

### Treatment

Treatment for measles is primarily supportive care. Hydration and antipyretics, such as in other viral illnesses, are the mainstays of therapy. If a secondary bacterial infection such as otitis media or pneumonia develops, appropriate antibiotics are indicated. Vitamin A is a measles-specific treatment that is critical in any patient with low levels; it can be administered orally.^5^ This treatment can lessen severity and even prevent mortality. The dose of vitamin A for measles patients is large, typically 200,000 international units (IU) for two days (50,000 IU if under 6 months and 100,000 IU for 6–11 months).^9^

Measles is a major cause of death in refugee camps where vitamin A deficiency is common, immunity is poor, and conditions are crowded. For every case of measles, 50 more persons in the camp are thought to be incubating disease. Vitamin A supplementation significantly reduces mortality.

### Prevention

The vast majority of cases of measles in patients with intact cellular-mediated immunity are uncomplicated and resolve by about 7–10 days after onset of illness. In fact, in prevaccination times, mothers used to purposely expose their children so they would all get sick at once and develop immunity before adulthood. Nevertheless, in modern days, disease prevention should be the goal.

All persons should be vaccinated against measles (as part of the measles, mumps and rubella [MMR] vaccine) unless there is a medical contraindication to live virus immunization. Vaccination is highly effective in preventing disease, but it is not 100% protective, even in persons who have received two doses of vaccine at appropriate intervals. Also, some patients are too young to be vaccinated, i.e., those under 12 months of age.

There has been a movement in the U.S. by some parents to avoid or delay vaccination based on personal, philosophical beliefs that are not medical or religious in origin. While this is permitted in some states, it results in decreased herd immunity, placing persons with medical contraindications to vaccination and others at greater risk. This reluctance to vaccinate is based in part on a 1998 study that reportedly found a link between the MMR vaccine and autism.^10^ This study has been discredited, the researcher accused of providing fraudulent data, and the paper has been retracted.

#### Post-Exposure Prophylaxis and Precautions

Infants under six months of age and nonimmune/nonimmunized pregnant women, if exposed, should receive passive immunity with intramuscular immune serum globulin. When administered within six days of virus exposure, these antibodies can prevent measles or reduce illness severity. If they are over six months, infants should receive a standard vaccination. Other nonimmunized persons who are exposed to measles should receive vaccination as well, ideally with 72 hours of the exposure. While vaccination might not prevent the disease, if illness develops it is generally less severe and for a shorter duration than in completely unvaccinated persons.

Household contacts should be advised that measles is highly contagious and infected family members should be isolated from four days before to four days after the rash manifests. Anyone at risk who is not fully vaccinated should receive vaccine as soon as possible. Most people born or living in the U.S. before 1957 have had measles and are therefore immune.

### Disposition

As with any other patient presenting to the ED, it is important for EPs to be familiar with admission vs. discharge criteria. Admission criteria for measles patients are similar to those for others. However, there are special considerations for patients being discharged. As measles is highly contagious, there are public health considerations as well as individual concerns for patients who do not meet hospitalization parameters. Infected patients must be isolated from others and public health must be notified so that contact tracing and community protective measures can be instituted.

EPs should provide return precautions. In addition to routine instructions, these should include the following: 1) monitor for occurrence of rash if one is not already present, and 2) pay special attention to the development of respiratory distress and neurologic symptoms. For someone being discharged, clinicians should document that the patient is well nourished and that the family is not in poverty (therefore not suspected to be Vitamin A deficient), and that there are no neurological or respiratory risks.

### Identify-Isolate-Inform

**Figure 6:**
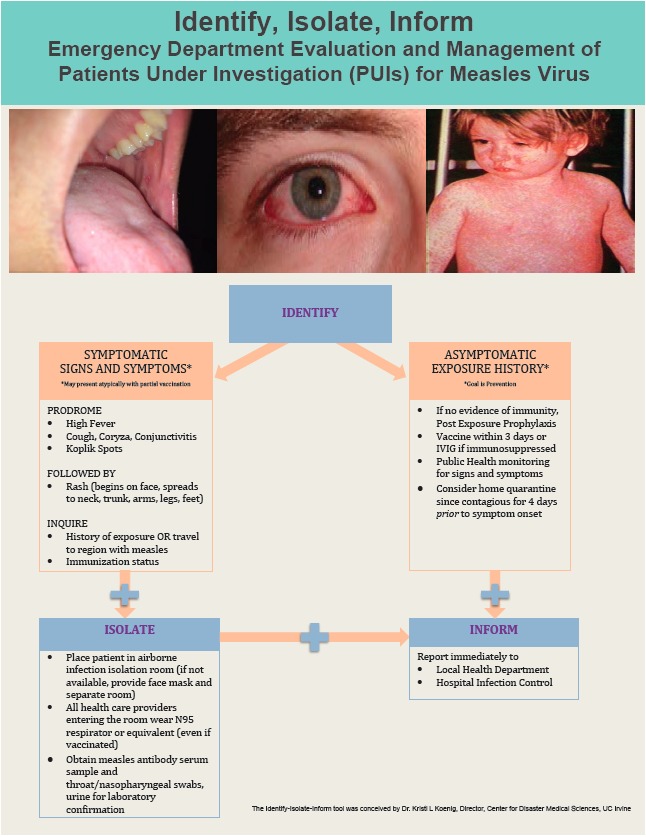
Identify, isolate, and inform tool. *IVIG*, intravenous immunoglobulin

The Identify-Isolate-Inform tool initially developed for Ebola virus disease^4^ can be modified for the ED evaluation and management of patients under investigation for measles virus (Figure 6). While patients typically present to the ED with symptoms, during an outbreak, concerned but asymptomatic patients and parents of potentially exposed children may seek care. Therefore, the first branch of the algorithm involves determination of whether the patient is symptomatic or asymptomatic. For asymptomatic patients, the goal is prevention of disease in both the individual and the population. This is accomplished by assessing exposure history and patient risk and, if it exists, providing post-exposure prophylaxis (with vaccine or with immune serum globulin if the patient is immunocompromised).^11^ The patient must then undergo public health monitoring for 21 days to monitor for the development of signs and symptoms. Home quarantine should be strongly considered, as patients with measles may be contagious for a few days prior to onset of symptoms.^12^

Conversely, patients with signs and symptoms of measles (prodrome of fever, cough/coryza/conjunctivitis, Koplik spots followed by rash), should be immediately masked and isolated using airborne precautions. To assist in risk assessment, EPs should inquire about immunization status, sick contacts, and travel to a region with measles. All healthcare providers, including those who have been vaccinated, should don N95 respirators or equivalent respiratory protection prior to caring for suspected measles patients. As with other airborne diseases, clinicians must be current on their “fit testing” requirements (typically renewed annually) in order to properly use an N95 respirator. Isolated patients should have samples obtained urgently and sent to the local public health department laboratory for disease confirmation.

Whether patients are symptomatic and immediately isolated or asymptomatic and exposed/at risk, public health authorities must be immediately notified 24/7. In addition, clinicians should promptly inform the hospital infection control and prevention practitioner on duty, regardless of time of day.

## CONCLUSION

**Figure 7:**
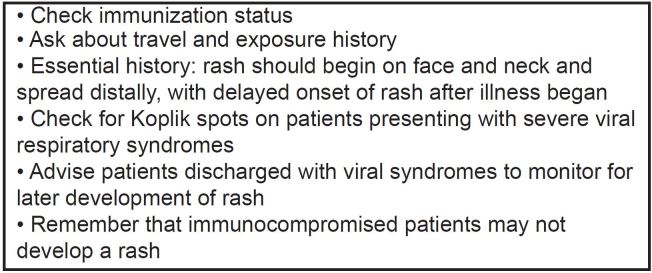
Measles pearls.

Measles is a highly contagious preventable viral disease that had been declared eradicated in the U.S., but made a resurgence starting in 2014. Identify-Isolate-Inform is a tool for EPs to apply when patients who may have measles present to the ED. In addition, several pearls are helpful when managing measles patients (Figure 7). As emergency physicians working on the front lines of clinical medicine, we must be prepared for emerging and reemerging infectious diseases.
